# A study of the impact of digital technology on industrial ecologisation in the Yellow River Basin of China

**DOI:** 10.1038/s41598-023-49933-1

**Published:** 2023-12-18

**Authors:** Qinqin Xu, Siliang Shu

**Affiliations:** 1 School of Economics, Shanxi University of Finance and Economics, Taiyuan, China; 2 School of Economics, Management and Law, Jiangxi Science and Technology Normal University, Nanchang, China

**Keywords:** Environmental economics, Urban ecology, Ecology

## Abstract

The Yellow River Basin is an important ecological barrier and economic core area in China, with problems such as fragile ecological environment and ecosystem degradation, and promoting industrial ecological transformation in resource cities is an important way to protect and improve the ecological and logical environment of the Yellow River Basin. Using panel data of 35 resource-based cities in the Yellow River Basin from 2012 to 2021, the impact of digital technology on industrial colonisation is empirically explored. The study finds (1) digital technology has a driving effect on the industrial ecological transformation of resource-based cities in the Yellow River Basin, and can be a new production tool to stimulate economic vitality; there is obvious regional heterogeneity in the impact of digital technology on industrial ecology, which significantly promotes the industrial ecological transformation of mid-stream and declining resource-based cities, and the facilitating effect is more obvious for declining resource-based cities. (2) From the moderating effect, fiscal decentralisation positively moderates the non-linear relationship between digital technology and industrial ecology. (3) From the perspective of threshold effect, the impact of digital technology on industrial ecologisation has a double threshold effect based on fiscal decentralisation, i.e. at the early stage of digital technology development, a reasonable degree of fiscal decentralisation can significantly promote the industrial ecological transformation of resource cities, but after the development of digital technology to a certain extent, the impact of fiscal decentralisation on industrial ecologisation will be gradually weakened, and will even bring negative effects to industrial transformation. Therefore, improving the development system of digital technology, giving the government moderate financial autonomy, and at the same time adhering to the local conditions and exploring the ecological development road in line with the characteristics of resource cities in the Yellow River Basin have positive significance for the industrial ecological transformation of resource cities in the Yellow River Basin.

## Introduction

To promote high-quality economic development, we should focus on promoting industrial ecology. As China’s important strategic guarantee base for energy resources, resource cities in the Yellow River Basin are rich in coal, oil, natural gas, and non-ferrous metal resources, and are China's important energy, chemical, raw materials, and basic industrial bases, and have a pivotal strategic position in the overall situation of socialist modernization. In December 2013, the State Council formally enacted the “National Plan for Sustainable Development of Resource Cities (2013–2020), the transformation of resource cities has shifted from solving historical legacy problems to economic, ecological, and social benefits. There are a total of 54 resource cities along the Yellow River, accounting for 43% of the total number of resource-based prefectural-level administrative districts in the country, making it a relatively concentrated area of resource cities in China. In addition, as the center of gravity of China's energy production and supply continues to shift to the central and western regions, some resource cities are forced to reach a higher degree of industrialization, resulting in a lack of technical support for the conversion of production, learning, and research, and other difficulties constraining the resilience of resource cities in the Yellow River Basin to weaken, and an imbalance in industrial development occurs.2022 In October 2022, the 20th CPC National Congress put forward the proposal “Promoting ecological protection and high-quality development of the Yellow River Basin” in its report. In October 2022, the report of the 20th CPC National Congress proposed “promoting ecological protection and high-quality development in the Yellow River Basin”, which put forward new and higher requirements for the industrial ecological development of resource cities in the Yellow River Basin. Combined with the Ruhr area in Germany, Lorraine in France Houston in the United States, and other resource-based cities in the face of resource depletion and the problem of a single industrial structure to adjust the employment structure and industrial structure as the focus of structural optimization of the basic completion of the industrial transformation, the transformation and development of our country is much better than. Although successful cases and theories can be learned from abroad, due to different national conditions, different policy systems, and resource environments, industrial transformation methods cannot be copied. Therefore, transforming resource cities in the Yellow River Basin into industrial ecology has become the key task of China’s current economic development.

As a result, this paper analyses the development status quo and ecological transformation path of resource cities in the Yellow River Basin by analyzing the relevant data of 35 resource cities in the Yellow River Basin, based on the perspective of digital technology, and uses the development of digital technology to boost the digital development of resource cities in the Yellow River Basin, which has an important practical and theoretical value for boosting the balanced development of resource cities’ industries. At the realistic level: first, based on the current situation of industrial structure development of resource cities in the Yellow River Basin, it accelerates the transformation of industrial structure to greening and colonisation with the help of digital technology means such as big data, internet, artificial intelligence, etc., and improves the development resilience of resource cities. Secondly, it provides a reference for the national resource cities to promote the transformation of industrial structure to ecology through the development of digital technology level, to realize the stage-by-stage goal of the ecological transformation of the industrial structure of the national resource cities. At the theoretical level: first, to expand the scope of application of the theory of digital technology supporting the transformation of resource-based cities towards ecologisation, and to enrich the relevant theoretical results of digital technology-driven ecologisation transformation of resource-based cities. Secondly, by studying the impact of digital technology on the ecological transformation of resource cities in the Yellow River Basin, we try to analyze the new opportunities faced by resource cities in the Yellow River Basin in their ecological transformation in the era of digital technology from the perspective of fiscal decentralization and summarise the findings to enrich the theories related to digital technology and industrial colonisation. The possible marginal contributions are: firstly, in terms of research perspective, focusing the research object on resource cities in the Yellow River Basin with serious resource dependence problems and difficulties in industrial ecologisation from the research perspective of digital technology, collecting panel data on digital technology, fiscal decentralisation, etc., to provide more reasonable and reliable empirical data for the transformation of industrial structure to ecologisation; secondly, in terms of research methodology, given that there is no consistent viewpoint on digital technology’s indicator construction, this article still follows the paradigm of ex ante estimation, constructs the indicator system of digital technology for resource cities in the Yellow River Basin, and invokes fiscal decentralisation to construct the threshold model and the adjustment model, to measure the impact mechanism of digital technology on industrial ecologisation more scientifically, and expects to be able to reveal some new important discoveries and obtain a brand new dimension of empirical interpretation; thirdly, in terms of the depth of the research, the article starts from digital technology, fiscal decentralisation and industrial ecologisation, the article clarifies the theoretical mechanism of digital technology and industrial ecologisation in resource cities, and further explores the heterogeneous impacts of digital technology on the transformation of industrial ecologisation from the perspectives of upstream, midstream and downstream, as well as the four types of growth, maturity, decline and regeneration, so as to provide a theoretical reference for the study of enhancing the urban economic resilience of domestic and foreign resource cities. The study will provide a theoretical reference for the study of enhancing urban economic resilience of resource cities at home and abroad.

## Literature review and critique

The industrial ecology of resource cities is related to the realization of the goal of high-quality economic development in the Yellow River Basin and is also the key to alleviating the pressure of resource mismatch. The Blue Book of Ecological Governance: Report on the Development of Ecological Governance in China (2020–2021) points out that the ecological status of the Yellow River Basin is related to the ecological security of North China, Northwest China, and even the whole country. The Yellow River Basin has the largest area of ecological fragility and the most fragile ecological types, with the distribution of ecological fragility zones such as the compound erosion of the Qinghai-Tibetan Plateau, the intersection of a desert oasis in Northwest China, the intersection of agriculture and animal husbandry in the north, and the intersection of coastal land and water, how to implement the issue of ecological protection of the Yellow River Basin in the environmental protection work is particularly important. Due to the unbalanced development of resource cities, there are obvious regional differences in economic and digital development^1^, the impulse of some economically backward cities to sacrifice the ecological environment and pursue short-term economic growth still exists, resulting in the contradiction of “strong green water and green mountain base and weak ecological capital output”. The problem of industrial structure imbalance is serious. Resource cities in the Yellow River Basin have been carrying out high-intensity resource development and utilization for a long time, and most of the resource-based industries have serious environmental problems, and the industrial structure that favors labor-heavy industries deteriorates the ecological environment^2^, leading to serious overloading of the carrying capacity of the resources and the environment^3^. In particular, cities in the basin relying on coal and electricity resources for economic development have excessive energy consumption, reducing biodiversity and harming the ecological environment^4^, and ecological restoration is extremely difficult and slow, which restricts the sustainable development of resource cities and leads to imbalance in industrial structure^5^.

Digital technology has become the engine of industrial transformation and social change^6^, digital technology is the information technology spawned by the use of data, digital tools^7^, manifested by the Internet, numerical control technology and artificial intelligence and other representative of the emerging technologies constitute the technology system. Digital technology has the advantages of low interaction cost, rich communication content, easy access to information, etc. The penetration of digital technology truly reflects the economic dimension of “inclusiveness”^8^, providing technical support for the development of green economy, which is conducive to the sustainable development of the economy^9^.

Analysed in terms of economic scale, digital technology is integrated into economic development in a new mode^10^. Through the change in digital technology, the scale of China's digital economy will reach 50.2 trillion yuan in 2022, and in the same year, the scale of China's digital industrialization will reach 9.2 trillion yuan, and the scale of industrial digitization will be 41 trillion yuan, accounting for 18.3% and 81.7% of the proportion of the digital economy, respectively, to realize the subversion and reshaping of industrial development. Digital technology has deeply penetrated the traditional manufacturing industry, promoting the process of digital transformation of the traditional manufacturing industry^11^, which is beneficial to reducing the imbalance of regional economic development and empowering the high-quality development of the economy^12^. At the same time, the development of digital technology is conducive to the development of China’s Internet by leaps and bounds, narrowing the digital divide^13^, and continuously strengthening the foundation of digital economic development through the establishment of the platform economy, the sharing economy, and other new business development.

Analyzed in terms of economic efficiency, digital technology, as a means to improve the productivity, intelligence, and flexibility of traditional manufacturing processes^14^, has been widely used in industrial manufacturing-based industries and has achieved improved economic efficiency in industrial industries through the integration of digital economy industries with manufacturing-based industries. Empowering the production chain enhances the synergistic ability of urban development and achieves the improvement of the synergistic efficiency of all kinds of industries. Accelerating industrial agglomeration through deep integration with industries, changing the traditional production methods of products, and improving industrial economic efficiency^15^. In addition to this, scholars have analyzed digital technologies from the banking industry^16^, the healthcare industry^17^, and industrial enterprises^18^ that can transform low-level entities, such as data, into information and knowledge to product information transformation of services.

Regarding the research on industrial ecology, the earliest foreign research began in 1989, Frosch and Gallopoulos^19^ defined the concept of “industrial ecology” for the first time, pointing out that the use of technological innovation, so that the traditional industrial activities are gradually transformed into advanced industrial ecosystems. The earliest domestic research on the theory of industrial ecology usually places the industrial system within the ecosystem, explores the harmonious interaction between the industrial system and the ecosystem, analyses the process of energy exchange and material conversion between the systems, and maximizes the outputs under the conditions of limited natural resources^20^, but there are fewer researches on industrial ecology in the level of dynamic analysis.

To sum up, resource-based cities in the Yellow River Basin adopt unreasonable ways to develop reserve resources and rely on heavy industry to develop their economy, for which they pay the price of ecological environment deterioration and limited economic development. Scholars mostly study the application of digital technology in production and life, and there are fewer studies on the ecological development of the industrial structure of resource-performing cities by digital technology, and the studies on the industrial ecology of resource-forming cities in the Yellow River Basin are still with certain limitations and lack of planning suggestions for the transformation, and the studies on the selection of industrial ecological paths, the construction of indexes, and the measurement of ecological level based on the construction of the model need to be further strengthened. The research on the selection of industrial colonization path, the construction of indicators and the measurement of colonization level based on the model should be further strengthened.

To this end, taking the 35 prefecture-level cities in the Yellow River Basin as the research object, we try to promote the ecological transformation of the industry with digital technology as the main line, and comprehensively describe and deeply analyze the “three steps” of digital technology to reduce the pollution emission and promote the ecological development: firstly, by promoting the balanced development of the industry, which is an important innovation of the organizational form of the division of labor. Firstly, by promoting the balanced development of the industry, which is an important innovation in the organizational form of division of labor, it improves the development and collaboration efficiency of the industrial sector; secondly, by empowering the whole chain of production, digital technology enhances the synergistic ability of the whole domain and chain of business, and improves the synergistic efficiency of the industrial industry and the information technology platform; thirdly, through the in-depth integration of digital technology into the key areas of carbon emission, it reduces the consumption of energy and resources, and promotes the optimization of the traditional industry's energy and cost, so that energy conservation, cost reduction, and quality enhancement and efficiency can be achieved overall. Based on theoretical analysis, this paper applies the entropy weight method to measure the level of digital technology in resource cities, constructs a fixed-effect panel regression model, analyses the role of digital technology on the transformation of industries in resource cities in the Yellow River Basin into ecology, and explores the path of realizing the ecology of industries with the help of digital technology, to provide countermeasures and suggestions for the sustainable development of resource cities in the Yellow River Basin according to the results of the empirical test.

## Theoretical analysis and research hypothesis

**Figure 1:**
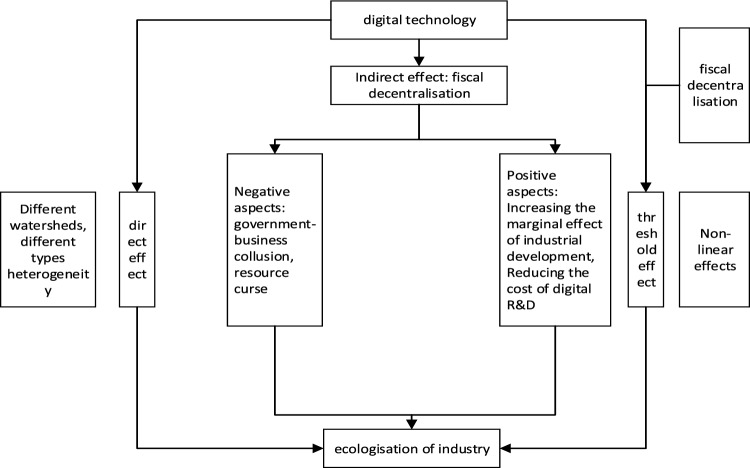
Analytical framework diagram.

Based on the industrial structure theory and the measurement of the fixed-effects panel regression model, we analyze the impact mechanism of digital technology on industrial ecology, the threshold and regulation mechanism of fiscal decentralization and digital technology on industrial ecology, and the multilayered perspective analysis of the heterogeneity test of digital technology on industrial ecology, and put forward the full text of the research framework (Fig. 1) and theoretical assumptions.

### The direct impact of digital technologies on the ecologisation of industrial industries

Under the guidance of industrial structure theory, resource cities in the Yellow River Basin should pay attention to the quality and quantity of industrial development while focusing on resource development and utilization in the process of industrial ecological transformation. Through the deep integration of digital technology and industrial development, digital empowerment strengthens the management of resource exploitation in resource cities in the Yellow River Basin, integrates digital technologies such as the Internet, artificial intelligence, and big data into the industrial ecological chain, product chain, and value chain, and promotes industrial change, to effectively reduce the consumption and destruction of the ecological environment by industrial development, grow the city’s economic efficiency, and achieve the ecological development of the city's industrial industry and economy. Ecological development of the city’s industrial industry and economy.

Summarising the existing research literature, it is found that digital technology is the power source for promoting ecological protection and the root of ecological civilization construction. In all fields of economic and social development, the importance of ecological environmental protection is becoming more and more prominent, increasing the strength of ecological environmental protection and creating conditions for the development of the industrial industry with a good ecological environment. The application of digital technology promotes information resource connection and sharing, smooth circulation of resource elements, and greatly enriches the theoretical basis of digital economics. Through the application of digital technology, industrial economic development gets a more convenient and open environment, accelerates inter-regional synergistic cooperation, and speeds up the transformation path of industrial industries. The research about digital technology boosting industrial ecology is analyzed from the following aspects:

From the perspective of development speed, China's digital technology has been in a steady and healthy state since 2013, and it is the right time to promote structural transformation with the help of new technologies, new digital elements, and other external forces in the development of new forms^21^. Digital technology to promote the digital transformation of traditional industrial industries and empower the industrial transformation and upgrading with digital technology will not only create new investment opportunities for the industrial development of resource-based cities but also promote industrial technological innovation and industrial change^22^, expand the production boundary, effectively alleviate the problem of rising labor costs, improve the industrial production efficiency and related products profitability level, the industrial economy quickly achieved the resumption of production and stable growth, fully demonstrating the great role of digital technology in enhancing the resilience, elasticity, and development speed of the industrial economy.

From the perspective of improving quality and efficiency, digital technology has empowered the industrial sector to save energy and reduce carbon emissions, and to improve industrial energy use efficiency and green manufacturing. Digital technology makes data a new production factor, and comprehensive penetration of industrial development promotes the overall change of the production mode of resource-based cities. Relying on the collection and calculation of digital technology, resource cities achieve precision in decision-making analysis, develop low-carbon products, and reduce industrial pollutant emissions; through the deep integration of digital technology and industrial development, reduce the development cycle and cost of green and low-carbon products to meet low-carbon demand; through the environmental pollutant emission system, carbon emission management system, etc., to achieve carbon emission verification and diagnosis, and help carry out targeted energy saving and emission reduction optimization to achieve industrial production and resource scheduling optimization, improve resource use efficiency, and achieve ecological economic integration of one, two and three industries^23^.

From the perspective of the development scale, on the one hand, digital technology relies on the virtual economy, penetrates various fields of industrial development, and prompts various industries to reach the development level of networking, digitization, and cloud; on the other hand, digital technology breaks the state of mutual separation between product production and consumption, integrates the production of industrial products in resource-oriented cities, optimizes the design of products and the injection of production capacity with the help of data analysis, and makes use of the industrial Internet platform to accurately match the user's demand, expanding the scope of consumption and improving effective supply.

Based on this, this paper puts forward hypothesis H1.

- H1: Digital technology effectively promotes the transformation of the industrial structure of resource-based cities in the Yellow River Basin towards ecology.

### Indirect effects of digital technology on industrial ecologisation

Under the current fiscal decentralization system and incentive assessment system in China, local governments will use financial means or administrative measures to intervene in economic development, attract and control the development and use of resources inside and outside the city, and the resulting resource barriers make the industrial structure disordered, and the phenomenon of industrial structure deviating from the local resource endowment occurs. For resource cities, scholars hold different opinions on the relationship between fiscal decentralization and industrial ecology.

Analyzed from a negative perspective: on the one hand, fiscal decentralization is not moderately decentralized, local governments will pursue higher socio-economic growth rates while neglecting expenditure inputs on environmental protection, education, and other public services, which is not conducive to environmental protection in resource cities. On the other hand, for resource cities rich in energy reserves, the pursuit of rapid economic growth, the emergence of political and corporate collusion, the formation of industrial structure in favor of industry, ignoring the protection of the ecological environment and the degree of damage, it is easier to fall into the resource curse.

Analysis from a positive perspective: in the context of the increasing integration of digital technology into the social economy, local governments must tend to the development of the digital industry in the role of financial decentralization system, accelerate the process of development of digital technology, integrate information through the basic digital platform based on the information industry, promote the efficient exchange of information, break down the barriers to information exchange, improve the behavior of government services, and support the development of digital technology to provide information transmission for the structural transformation. Support. Therefore, the degree of government financial decentralization affects the level of digital technology development, which is beneficial to accelerate the process of digital technology development, and then affects the industrial ecological transformation of resource-based cities. There are two reasons for this: first, improving the factor structure and configuration of fiscal input and output and optimizing the structure of digital factors can improve the marginal efficiency of industrial production factors; second, fiscal decentralization promotes the role of innovative factors such as talents, funds, and enterprises, reduces the transaction costs of digital factor flows and the marginal costs of digital R&D, thus enhancing the level of development of digital technology and providing a guarantee for the ecological transformation of resource-based cities. Provide guarantee. Thus, hypothesis 2 is proposed.

- H2: Fiscal decentralisation moderates the role of digital technologies for industrial ecologisation in resource-based cities.

### Non-linear threshold characteristics of digital technologies for industrial ecologisation

Because of the different incentive preferences of local governments under different levels of fiscal decentralization, the effect of fiscal decentralization on the balanced industrial structure of digital technology also shows dynamic evolution. When the level of fiscal decentralization is low, local governments are more inclined to achieve rapid economic growth in the short term, and fiscal decentralization will stimulate local governments to pursue political performance with the collusion of government and enterprises. Local governments pursue the development of productivity unilaterally, ignoring the controlled exploitation and efficient use of resources and environment, resulting in ecological damage to constrain the development of productivity, productivity through the development of natural predatory development of the vicious circle, which makes the local government pay more attention to the efficiency of the allocation of factors of production, and the expansion of the industrial scale has a promotional effect, which in turn improves the level of development of the regional industry, and will be more conducive to the development of the digital technology to play a role in the ecological structure of the industrial structure. Technology to play on the transformation effect of industrial structure ecological. When the level of fiscal decentralization reaches a certain degree, the local government has greater autonomy over financial expenditures, and may intervene excessively in the development of the regional industrial scale, and also has a stronger incentive to set up industrial barriers or resource exploitation, but will not be conducive to the improvement of digital technology to improve the balance of industrial structure. Based on this, this paper proposes hypothesis 3.

- H3: There is a threshold effect based on the degree of fiscal decentralisation of the impact of digital technology on industrial ecologisation.

### Heterogeneity analysis of digital technology-enabled industrial ecological transformation

It is of great practical significance to promote the green development of industries in the Yellow River Basin through the initiatives of industrial structure adjustment and layout, green optimization, and transformation of traditional industries to achieve the synergistic promotion of carbon reduction, pollution reduction, green expansion and growth of industries in the Yellow River Basin. Analysed from different resource-type cities, most resource-type cities have the status quo of a high proportion of traditional industrial industries. Given the different resource cities’ resource reserves, digital infrastructure, and digital technology industries, there are differences in the level of digital technology and differences in the promotion of industrial industry development in resource cities. From the perspective of resource endowment analysis, the upstream area, as an important resource energy and heavy chemical industry base in China, has the dilemma of weak economic strength, which cannot provide good development conditions for ecological transformation; while the middle and lower reaches of the region can rely on sound infrastructure, a good economic foundation, gather high-quality labor force and advanced technology, and drive the benign cycle of the economy, which provides a platform for the advantages of digital technology development. Different types of resource-based cities in the Yellow River Basin differ in resource endowment, economic development, and other aspects, and the role of digital technology is affected by many factors, with obvious regional heterogeneity. Therefore, this paper proposes hypothesis H4.

- H4: There is heterogeneity in the impact of digital technology on industrial ecologisation in resource-based cities in the Yellow River Basin.

## Research design

### Research methodology

To analyze the influence mechanism of digital technology to boost the industrial colonization of resource cities in the Yellow River Basin, based on the above theoretical analysis of industrial structure, a panel data analysis method is used to construct a fixed effect panel regression model, and further construct a moderating model, a threshold model, and a robustness test. Industrial colonization is taken as an explanatory variable, and the influencing factors are analyzed from four perspectives, i.e. the level of economic development, the intensity of industrial carbon emissions, the level of industrialization, and the level of human capital. Digital technology is the core explanatory variable, and regression analysis is conducted using panel data of 35 resource cities in the Yellow River Basin to explore the degree of influence of each factor on industrial colonisation in resource cities.

### Modelling

To analyse the effect of digital technology on the ecologisation of the forestry industry, the following model is constructed:

1 $$Eco_{\text{it}} = \partial_0 + \partial_1DITE_{\text{it}} + \partial_ 2COTL_{\text{it}} + \varepsilon_{\text{it}},$$

Due to the relative increase of observations, the fixed-effects regression model can get the parameter consistent estimation value, at the same time, the panel data modelling can get more dynamic information than the single cross-section data modelling, so the fixed panel model is used. Where: Ecoit is the industrial ecological status of resource-based areas in the Yellow River Basin for city i in year t, i represents the region, and t represents the period; DITEit denotes the level of digital technology development in each resource-based city; COTL denotes the control variables, including the economic development level (EDL), the carbon emission intensity (CAR), the level of industrialization (INL) and the level of human capital (HCL) in four perspectives; εit denotes the random perturbation term.

This paper adds the cross-multiplier term of digital technology development index and fiscal decentralization to model (1), to establish model (2) to verify the moderating effect of fiscal decentralization on the relationship between digital technology to promote industrial colonisation in resource-based cities.

2 $${{\text{Eco}}}_{{\text{it}}}={\partial }_{0}+{\partial }_{1}{\text{DITE}}_{{\text{it}}}\times {\text{DFD}}+{\beta }_{1}{\text{COTL}}_{{\text{it}}}+{\varepsilon }_{{\text{it}}}.$$

Considering the possible lag in the impact of digital technology on industrial colonization, this paper establishes a dynamic panel model with explanatory variables lagged by one period. Based on model (1), digital technology (DITE) lagged one period as an explanatory variable for regression test to alleviate the possible problem of reverse causality, see model (3).

3 $$Eco_{it} = \partial_0 + \partial_1DITE_{i,t - 1} + \partial_2COTL_{it} + \varepsilon_{it},$$

The panel threshold model, as an econometric model for analyzing whether economic parameters undergo sudden structural changes, can verify the relationship between fiscal decentralization and industrial ecologisation in the cities of the Yellow River Basin, and calculate the threshold value that affects the transformation of industrial ecologisation. Some resource-based cities are still dominated by heavy industry, with prominent structural problems such as the large proportion of traditional industries, relatively slow development of new industries, and imbalance of industrial weights, and the industrial structure has not gotten rid of the resource-dependent characteristics to a certain extent, only after the implementation of appropriate fiscal decentralization measures, the establishment of a fiscal transfer system to compensate for the historical arrears of resource-based cities, and the fostering of the development of resource-based cities to develop low-consumption, low-emission and efficient green industries. Therefore, to further reveal the difference in the effect of digital technology on the colonisation of urban industries in the Yellow River Basin under different fiscal decentralization measures, a threshold regression model is constructed. When there is a single threshold, the model is constructed as follows:

4 $$Eco_{it} = \alpha_0 + \sum\limits_{j = 1}^{n} {\alpha_1control_{jit} + \beta_1DITE_{it}I\left( {\chi \le \gamma_1} \right)} + \beta _2DITE_{it}I\left( {\chi > \gamma_1} \right) + \varepsilon _{it},$$

When there is a double threshold, the model is constructed as follows:

5 $$Eco_{it} = \alpha_0 + \sum\limits_{j = 1}^{n} {\alpha _1control_{jit} + \beta_1DITE_{it}I\left( {\chi \le \gamma_1} \right)} + \beta_2DITE_{it}I\left( {\gamma_1 < \chi < \gamma _2} \right) + \beta_3DITE_{it}I\left( {\chi > \gamma_2} \right) + \varepsilon_{it},$$

where i denotes the province, t denotes the year, Eco is the explanatory variable (industrial colonization), X denotes the threshold variable fiscal decentralization, DITE denotes the explanatory variable digital economy, α1 and β1 are the coefficients, and γ is the threshold value. In this paper, when the threshold variable χ is fiscal decentralization, the core explanatory variable DITE is digital technology. control denotes the control variable and ε denotes the random error term.

### Variable selection

#### Explanatory variable

Resource-based cities are generally old industrial cities that have prospered due to resource exploitation. Due to irrational resource exploitation industrial development imbalance and other factors, a large amount of pollutants have been emitted in the process of industrial development, leading to ecological imbalance and irreversible environmental impacts. Therefore, it is crucial to promote industrial transformation and upgrading, kinetic energy successive conversion, ecological restoration, and governance of resource cities with the help of digital technology.

The explanatory variable is industrial ecology (Eco), to reflect the ecological attributes of industrial development, referring to the studies of Li Bin et al.^24^ and Yin Baoqing^25^, we adopt MAXDEAP6.0 software and choose the two-way model with input priority to measure industrial ecology of resource cities in the Yellow River Basin. Each city was taken as a research unit, and the input indicators were labour, capital stock and energy consumption; labour was expressed by the labour population using the number of employees in urban units, capital stock was estimated by the perpetual inventory method, and energy consumption was expressed by the summed water supply and the whole society’s electricity consumption. Desired output is expressed by urban economic output, in which gross regional product and total retail sales of consumer goods are selected as elements of economic output, and non-desired output is expressed by industrial fume (dust) emissions, industrial sulphur dioxide emissions and industrial wastewater emissions. The measurement model is shown in (6).

6 $$Eco_{it} = \frac{1}{2}\left( {\frac{{(E_{it} - \partial_{{_{ei,it} }}^{*} E_{it} )/(G_{it} + \partial_{{_{g,it} }}^{*} G_{it} )}}{{E_{it} /G_{it} }}} \right) + \frac{1}{2}\left( {\frac{1}{3}_{U = ww + fu + so2} \sum {\frac{{(U_{it} - \partial_{{_{U,it} }}^{*} )/(G_{it} + \partial_{{_{g,it} }}^{*} G_{it} )}}{{U_{it} /G_{it} }}} } \right),$$

where α*ei, α*g, α*ww, α*fu, and α*SO_2_ are the optimal solutions of the super-efficient model.

#### Core explanatory variables

Based on the theory of information economics, data informatization is one of the core elements of the development of digital technology, and the development of resource cities can be organized and managed with the help of digital technology to maximize the degree of information availability and build a new stage of resource city development in the Yellow River Basin. The level of digital technology development (DITE) is mainly measured from three dimensions: digital input, digital environment, and digital output. Due to the study of the industrial ecological transformation of resource cities in the Yellow River Basin, this paper uses the total amount of green patents granted to measure the digital output, integrate green development into all aspects of the whole chain of industry, promote the green transformation of traditional industrial industries, and advancing the development of industrial ecology. The construction of indicators is shown in Table 1. The first step first homogenizes the raw data, and the second step applies the entropy weight method to the above measurements to obtain the digital technology index.

**Table 1:** Indicator system for comprehensive measurement of digital technology.

Objective layer	System level	Indicator layer	Indicator measurement	Attributes
Digital technology indicators	Numbers inputs	Internet penetration	Number of internet broadband accesses taken in pairs (thousands)	Positive
		Urban infrastructure development	Urban road space per capita (per cent)	Positive
		Level of digital inclusive finance	Ali research institute report and Peking University’s digital financial inclusion indicator data taken in logarithms	Positive
	Numbers environment	Industrial development environment	Drawing on Liu Huiwu et al.^26^ measurements	Positive
		Vitality of market entities	Number of business registrations in the current year (10,000 people)	Positive
		Entrepreneurial activity	Number of new enterprises per 100 people (10,000)	Positive
	Figures outputs	Patent grant	Total number of green patents granted (units)	Positive

#### Control variable

This paper analyses the influencing factors of industrial colonization in resource-based cities mainly from four perspectives: economic development level (EDL), carbon emission intensity (CAR), industrialization level (INL), and human capital level (HCL).

(1) Economic development level (EDL). Reflecting the level of socio-economic phenomena in different periods, it is an important symbol indicating the state of the regional economy and reflecting the potential for development, and is expressed in terms of gross regional product; (2) Carbon emission intensity (CAR). The amount of carbon dioxide emissions in the form of greenhouse gases released by the industrial production process in a region, expressed in logarithmic terms; (3) Industrialisation level (INL). Expressed as the share of the secondary sector in GDP, in percentage terms; (4) Human capital level (HCL). The literacy level and knowledge skills of the labor force in the region is one of the important factors affecting the success of industrial structure transformation. Expressed as the number of population with general undergraduate degrees and above.

#### Moderating and threshold variables

To further explore the role played by digital technology in the industrial colonization of resource-based cities, fiscal decentralization is introduced as a moderating and threshold variable. It is expressed by fiscal decentralization (DFD), which is derived from the ratio of fiscal budget expenditure to fiscal budget revenue. The non-linear role of fiscal decentralization on industrial colonisation through digital technology is further clarified. See Table 2 for descriptions of specific variables.

**Table 2:** Description of variables.

Type	Variable name	Symbol	Indicator measure
Explained variables	Ecological industrial structure	Eis	DEA measurement
Core explanatory variables	Digital technology composite index	DITE	Measured by entropy weight method based on relevant data
Control variables	Economic development level	EDL	Gross regional product (RMB billion)
	Carbon emission intensity	CAR	Emissions of carbon dioxide released in the form of greenhouse gases from industrial production processes (take logarithm)
	Industrialisation level	INL	Secondary industry/GDP (per cent)
	Human capital level	HCL	Population of general undergraduates and above (10,000 people)
Moderating variables/threshold variables	Fiscal decentralisation	DFD	Ratio of fiscal budget expenditure to fiscal budget revenue

### Data sources and descriptive statistics

This paper utilizes the 2012–2021 China Urban Statistical Yearbook, the 2012–2021 China Environmental Statistical Yearbook, China Energy Statistical Yearbook, China Environmental Yearbook of each prefecture-level city, and the 2012–2021 National Economic and Social Development Statistical Bulletin of 35 resource-based cities. Some missing data in the text were supplemented by interpolation. The empirical data and descriptive statistics are shown in Table 3.

**Table 3:** Descriptive statistics for variables.

Variable	Observations	Mean	Standard deviation	Minimum value	Maximum value
Eis	350.000	1.014	0.047	0.813	1.185
DITE	350.000	0.186	0.111	0.028	0.633
EDL	350.000	1620.416	1235.392	207.8152	5447.123
CAR	350.000	6.702	0.430	5.254	7.362
INL	350.000	0.504	0.113	0.156	0.758
HCL	350.000	4.277	4.596	0.033	24.202
DFD	350.000	3.051	1.621	1.171	8.453

As we know from Table 3, the standard deviation observations of the variables have a large degree of dispersion, indicating that the level of development of resource cities is uneven and varied, and it is necessary to analyze regional heterogeneity.

## Analysis of empirical results

### Analysis of baseline effects

According to the empirical results (see model I in Table 4), when digital technology is applied to the industrial development of resource cities, it has a promoting effect on the industrial structure towards ecology. The correlation coefficient between the two is 0.186, which passes the test at the 10% level, indicating that digital technology effectively stimulates resource cities in the Yellow River Basin to scientifically exploit resources, develop industries, reduce carbon emissions, and effectively promote the transformation of industries into ecology through its characteristics such as unique economic attributes and development potential. Hypothesis H1 is confirmed, and the overall regression results are in line with expectations.

**Table4:** Estimated results.

Modelling	Modellin 1	Modellin 2	Modellin 3
Variant	Eis	Eis	Eis
DITE	0.186* (0.103)		
DITE		0.266* (0.122)	
DICE*DFD			0.073* (0.034)
EDL	0.000* (0.000)	0.000* (0.000)	0.000* (0.000)
CAR	0.035* (0.020)	0.028 (0.022)	0.032 (0.020)
INL	0.126* (0.055)	0.161* (0.064)	0.142* (0.056)
HCL	0.004* (0.003)	0.005 (0.004)	0.005* (0.003)
Constant	0.702*** (0.136)	0.720*** (0.145)	0.734*** (0.136)
Sample size	350	315	350
Goodness of fit	0.052	0.058	0.068

"*", "**", and "***" indicate that the regression results are significant at the 10%, 5%, and 1% levels, respectively, and the values in parentheses are standard deviations. following table is the same.

The reasons for this are, firstly, the development of digital technology is conducive to the cultivation of new industries integrating digitalization and eco-environmental protection, and the promotion of eco-safety, energy-saving and environmental protection and other digitalized industrial projects with the orientation of green and low-carbon transformation; secondly, the application of digital technology promotes the development of industrial industries in resource cities in the direction of energy-saving and eco-environmental protection, aids in the digitalization of industries, and eases the dilemmas in the process of industrial development such as the reduction of pollution, the lowering of emissions, and the restoration of the environment; and, thirdly, the development of digital technology helps resource cities to build an ecological industrial chain that works together in terms of economic benefits and social benefits to realize a good circle of ecologisation of industries.

In addition, the regression coefficient of carbon emission intensity is significantly positive, indicating that the process of industrial ecological transformation must follow the inherent law of pollution reduction and carbon reduction. The regression coefficient of the industrialization level is significantly positive, indicating that the problem of industrial ecological transformation is accompanied by the industrialization process. The regression coefficient of the human capital level is significantly positive, indicating that the enhancement of human capital is beneficial to raising the public's awareness of environmental protection, abandoning the traditional industrial civilization, practically transforming the industrial development mode, and promoting the improvement of environmental quality and that the input of human capital is the result of the transformation of resource input, with the characteristic of increasing marginal returns in the long term.

### Analysis of moderating effects

Further discussion considers the deep integration of digital technology and fiscal decentralization and the real need to fully exploit the industrial structure transformation of resource-based cities in the Yellow River Basin. To this end, fiscal decentralization is introduced to capture the moderating effect on industrial colonisation through the cross-multiplier term of fiscal decentralization and digital technology, and the regression results are shown in Table 4, Model 3.

The results show that: the interaction term of financial decentralization R&D digital technology has a 10% confidence level promotion effect on the industrial structure of resource cities in the Yellow River Basin towards ecology, confirming the hypothesis H2. The possible reasons for this are: firstly, the decentralization of government affects the local government's financial investment in the ecological industry, and it has a guiding function for the social funds, which continues the policy support for digital technology. The orientation of the local government's green and low-carbon policy, the use of new technologies to transform and upgrade traditional industries, and the development of energy-saving and environmental protection industries have strengthened the low-carbon development of industries in resource cities and promoted the transformation of the industrial and economic structure to eco-industry. Secondly, fiscal decentralization, as an institutional factor, has a positive impact on the development of industries towards ecology. The development of digital technology provides a theoretical basis for the government's intervention, and digital finance plays a role in the positive externalities and public attributes such as eco-products of industrial ecology through the sharing of resources and promotes the development of new industrialization and new ecological development in resource-based cities in a coordinated manner. Thirdly, fiscal decentralization has obvious spillover effects. The government can better play the role of fiscal decentralization, encourage and support the development of digital technology, and guide the cumulative effect of ecological factors in industrial development, which has an important impact on strengthening the spatial linkage of industrial colonisation in resource-based cities.

### Analysis of threshold effects

To circumvent the effect of heteroskedasticity and avoid the outliers at both ends, the threshold variable removes 10% of the values at both ends and then conducts the test of the threshold effect, and after the bootstrap loop test for 300 times, the test results are listed in Table 5, which shows that there exists a threshold effect with fiscal decentralization as a two-tiered threshold, and the threshold values are 2.139 and 2.363, respectively, confirming Hypothesis 3.

**Table 5:** Threshold effect test results.

Threshold variables	Model estimates	Threshold estimates	F-value	P-value	Number of BS	Threshold		
DFD	Single threshold	Threshold value:2.139				10%	5%	1%
	Double threshold	Threshold value 1: 2.139	5.32*	0.107	300	19.899	24.680	28.794
		Threshold value 2: 2.363	8.82*	0.085	300	18.617	22.013	27.786

* indicates that the regression result is significant at the 10 per cent level.

The empirical results show that when the degree of fiscal decentralization is lower than 2.139, fiscal decentralization is negatively correlated with industrial colonization, i.e., fiscal decentralization inhibits the transformation of industrial colonization; when the degree of fiscal decentralization is in the interval of [2.139, 2.363], fiscal decentralization is positively correlated with industrial colonization, i.e., fiscal decentralization can promote the transformation of industrial colonization of resource cities significantly, and the coefficient of influence at this time is 0.175, which It means that for every 1 unit of government fiscal autonomy, the promotion effect on industrial ecological transformation is 0.175 units; when the degree of fiscal decentralization is higher than 2.363, the impact of fiscal decentralization on industrial ecological transformation is not significant. This indicates that when the degree of fiscal decentralization is higher than a certain degree, it will weaken the positive effect on the development of industrial colonisation. Through the above analysis, it can be seen that different degrees of fiscal decentralization will have a heterogeneous impact on industrial ecological transformation, and the two have an inverted *U*-shaped relationship. When the degree of fiscal decentralization is too low or too high, the promotion effect of fiscal decentralization on industrial ecological transformation will be weakened, and even have a negative impact, i.e. the financial autonomy given to local governments should be grasped in the "moderate range", and the financial autonomy should be controlled in a reasonable range, so that the financial funds can give full play to the leverage effect on the transformation of industrial ecological transformation of the resource-based cities. Leverage.

### Robustness check

In order to reduce the impact of possible endogeneity issues on the estimation results, one period lag is taken for all explanatory variables in the benchmark regression. Based on the empirical results, it can be seen that the results of the regression analysis are highly consistent with the original benchmark regression, indicating that the model is robust to a certain extent.

#### Endogeneity test

To explore whether there is a time lag effect in the impact of digital technology on industrial colonisation, the explanatory variables are taken one period lagged, and the regression results are shown in Table 4, Model II. As can be seen from the empirical results, the results of the empirical analysis of the lagged period are consistent with the benchmark regression. Based on endogenous considerations, digital technology with economic vitality and development resilience still significantly helps the industrial ecology of resource cities in the Yellow River Basin.

#### Heterogeneity test

In order to analyze the differences between different types of cities, this paper will classify the resource-based cities in the Yellow River Basin into four types: mature, declining, and regenerating and growing cities based on the National List of Resource-based Cities (2013). Based on the planning of "Comprehensive Planning of Yellow River Basin (2012–2030)", the resource-based cities in the Yellow River Basin are divided into upstream, midstream, and downstream areas, as shown in Table 6. The driving factors and internal action mechanism of digital technology on industrial ecology are explored, with a view to providing a basis and reference for formulating targeted, precise, and efficient transformation proposals for the structural transformation of different types of resource-based cities.

**Table 6:** City division.

Upstream	Midstream	Downstream	Growth	Mature	Declining	Regenerative
Ordos, Wuhai, Baotou, Zhangye, Jinchang, Wuwei, Baiyin, Shizuishan	Yangquan, Jinzhong, Changzhi, Jincheng, Yuncheng, Shuozhou, Datong, Xinzhou, Luliang, Weinan, Yulin, Yan'an, Qingyang, Pingliang, Baoji, Xianyang, Tongchuan, Linfen, Jiaozuo, Pingdingshan, Sanmenxia, Luoyang	Puyang, Hebi, Zibo, Jining, Tai'an	Ordos, Wuwei, Shuozhou, Yulin, Yan'an, Qingyang, Xianyang	Yangquan, Jinzhong, Changzhi, Jincheng, Yuncheng, Jinchang, Datong, Xinzhou, Luliang, Weinan, Pingliang, Baoji, Linfen, Pingdingshan, Sanmenxia, Hebi, Jining, Tai'an	Wuhai, Baiyin, Shizuishan, Tongchuan, Jiaozuo, Puyang	Baotou, Zhangye, Luoyang, Zibo

As shown in Tables 7 and 8. Heterogeneity of different resource-based cities in the Yellow River Basin, the validation concludes that the development of digital technology has a significant role in promoting the industrial ecology of midstream resource-based cities and declining resource-based cities, confirming hypothesis H4. The reasons are as follows: firstly, most of the cities with a high level of diversity of resource-based industries are located in the middle reaches of the river and are mainly concentrated in Shanxi, southern Shaanxi, and northwestern Henan, etc., and the upper reaches of the Yellow River Basin and the lower reaches of the resource-based The level of resource-based industrial path creation in the cities is relatively low, and the resource-based cities in the middle reaches are better endowed with resources, and it is easier for them to adopt digital technology to extend the industrial chain and develop the eco-economy on the basis of the existing resources. Secondly, for the declining resource cities that tend to deplete resources, lag behind in economic development, and have great pressure on the ecological environment, relying on digital technology to break the dualistic structure within the city, support the development of successive alternative industries, and enhance the city's ability to develop in a sustainable manner.

**Table 7:** A test for watershed heterogeneity.

Type (of object) variable	Upstream	Midstream	Downstream
	Eis		
DITE	− 0.115 (0.199)	0.635*** (0.147)	− 0.642* (0.239)
Control variables	Yes	Yes	Yes
Degree of fit	0.028	0.216	0.270
Sample size	80	220	50

**Table 8:** Type heterogeneity test.

Type (of object) variable	Mature	Growth	Regeneration	Decline
	Eis			
DITE	0.033 (0.248)	0.060 (0.198)	− 0.709* (0.286)	30.210*** (4.380)
Control variables	Yes	Yes	Yes	Yes
Degree of fit	0.051	0.274	0.446	0.940
Sample size	180	70	40	60

## Research findings and policy implications

This paper constructs a model of digital technology on industrial colonization, elucidates the impact of digital technology on industrial colonization from the theoretical level, and validates the inferences of the theoretical model by using the data of 35 resource cities in the Yellow River Basin from 2012 to 2021, obtains the research conclusions, and puts forward the policy insights.

### Findings

This paper measures digital technology and industrial colonization in 35 resource-based cities in the Yellow River Basin, and explores and analyses their transformation paths in depth, and draws three main conclusions. (1) From the perspective of the transformation path, digital technology has a significant role in promoting the transformation of industrial colonization, and there is heterogeneity across different river basins and types. Digital technology can significantly enhance the transformation of midstream areas and depleted resource cities to industrial ecology, and the promotion effect on depleted resource cities is more obvious. (2) From the perspective of regulating effect, digital technology increases the ecological environment management for cities by combining the degree of fiscal decentralization and then promotes the industrial ecological transformation. (3) The empirical research relying on the threshold model shows that there is a double threshold of fiscal decentralization. After the degree of fiscal decentralization crosses the threshold, the promotion effect of digital technology level enhancement on the positive adjustment of industrial ecological transformation increases.

### Policy implications

Combined with the research findings, this paper puts forward the following policy recommendations:

1. Focus on digital technology development and improve the digital technology development system. Give full play to the economic vitality of digital elements, integrate and practice the industry chain, artificial intelligence and other core technologies in the Internet with government governance, promote the development of industrial clusters in resource-based cities, so that industrial resources and digital technology platforms can be seamlessly connected and integrated to promote industrial development.
2. Optimise the fiscal relationship of resource-based cities and give the government moderate fiscal autonomy. The findings of this paper show that fiscal decentralisation can effectively promote the transformation of digital technology to industrial ecology. Therefore, the government should further adjust and improve the fiscal relations of resource cities, give full play to the information advantage and subjective initiative of local governments, improve the efficiency of resource allocation, and then promote the development of green economy.
3. Adhere to the local conditions and explore the ecological development path that meets the Yellow River resource cities’ characteristics. Most of the declining resource cities are diminishing returns to scale, improving the input of digital technology to find alternative industries, and taking the path of low-carbon economic development. At the same time, digital technology empowers upstream resource-based cities to implement major ecological protection and restoration projects to accelerate the curbing of ecological degradation trends; downstream resource-based cities focus on digital technology infrastructure, carry out comprehensive ecological environment improvement, and promote ecological protection and development. Strengthen the development of digital technology, cracking the resource-based cities that have long relied on energy and heavy industry, and the development of low-quality and low-efficiency problems.

From the experience of the ecological transformation of resource cities in the Yellow River Basin, it can be seen that digital technology effectively promotes the green development of industry, forming a virtuous cycle system of efficient resource extraction—product production and manufacturing—commodity circulation and consumption—waste recycling and disposal. Digital technology provides new possibilities for industrial transformation and economic development. Foreign resource cities should make good use of information technology, accelerate the construction of digital infrastructure, vigorously cultivate digital technology in the fields of cloud computing, big data, artificial intelligence, etc., promote the upgrading of digital industrial chain, make the economic structure more diversified, and provide important support and guarantee for the economy to achieve digital transformation.

## Data Availability

All data were obtained from the China Urban Statistical Yearbook, China Energy Statistical Yearbook, China Environmental Yearbook, and China Research Data Service Platform database (CNRDS).
